# Supplementary figures and images for: Comprehensive Time-Course Transcriptome Reveals the Crucial Biological Pathways Involved in the Seasonal Branch Growth in Siberian Elm (Ulmus pumila)
Source: Int J Mol Sci. 2023 Oct 7;24(19):14976. doi: 10.3390/ijms241914976 (PMC10573607; doi:10.3390/ijms241914976)

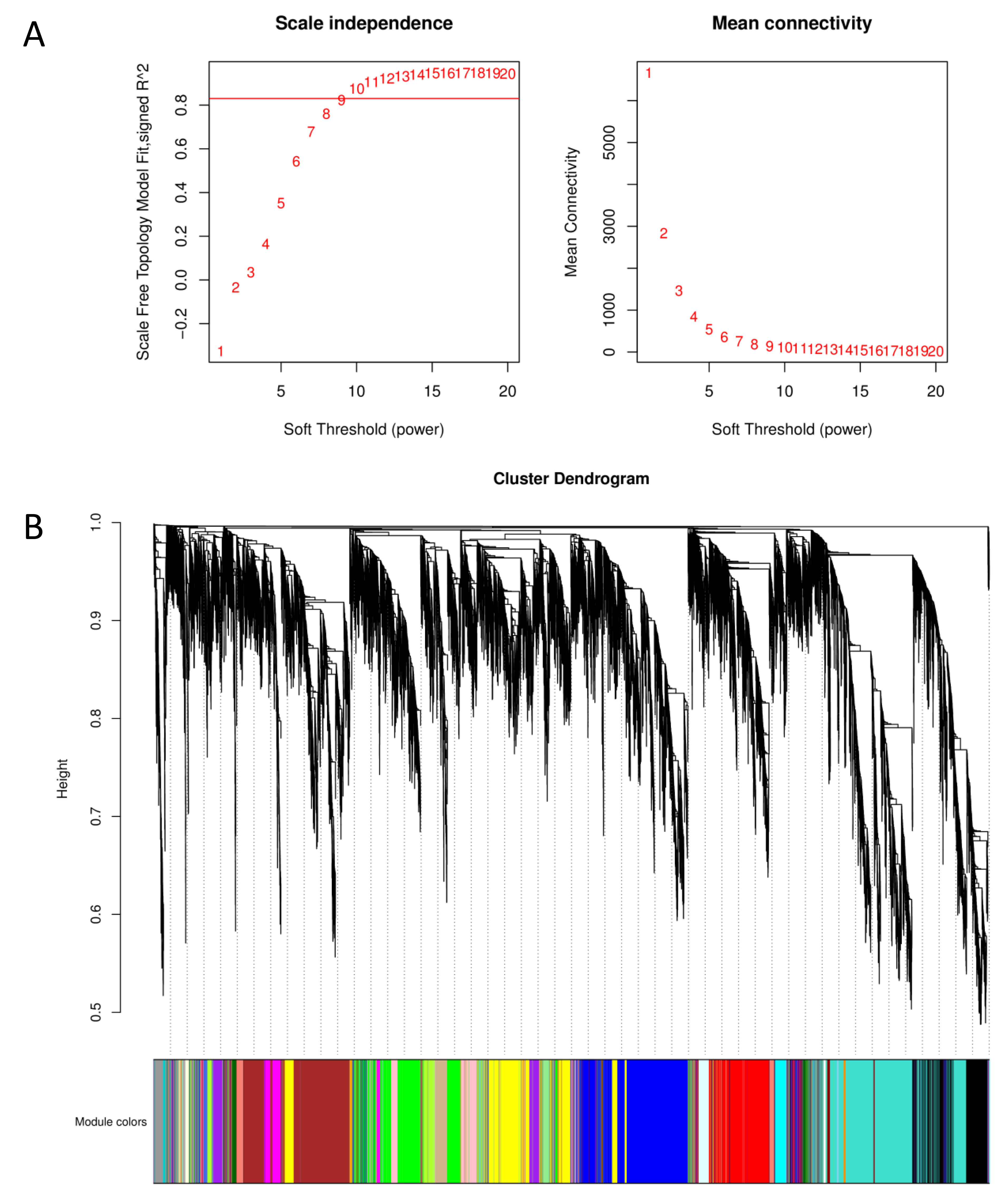

Supplement: Supplementary file 1 [file ijms-24-14976-s001.zip › Supplementary files/Figure S1.jpg]

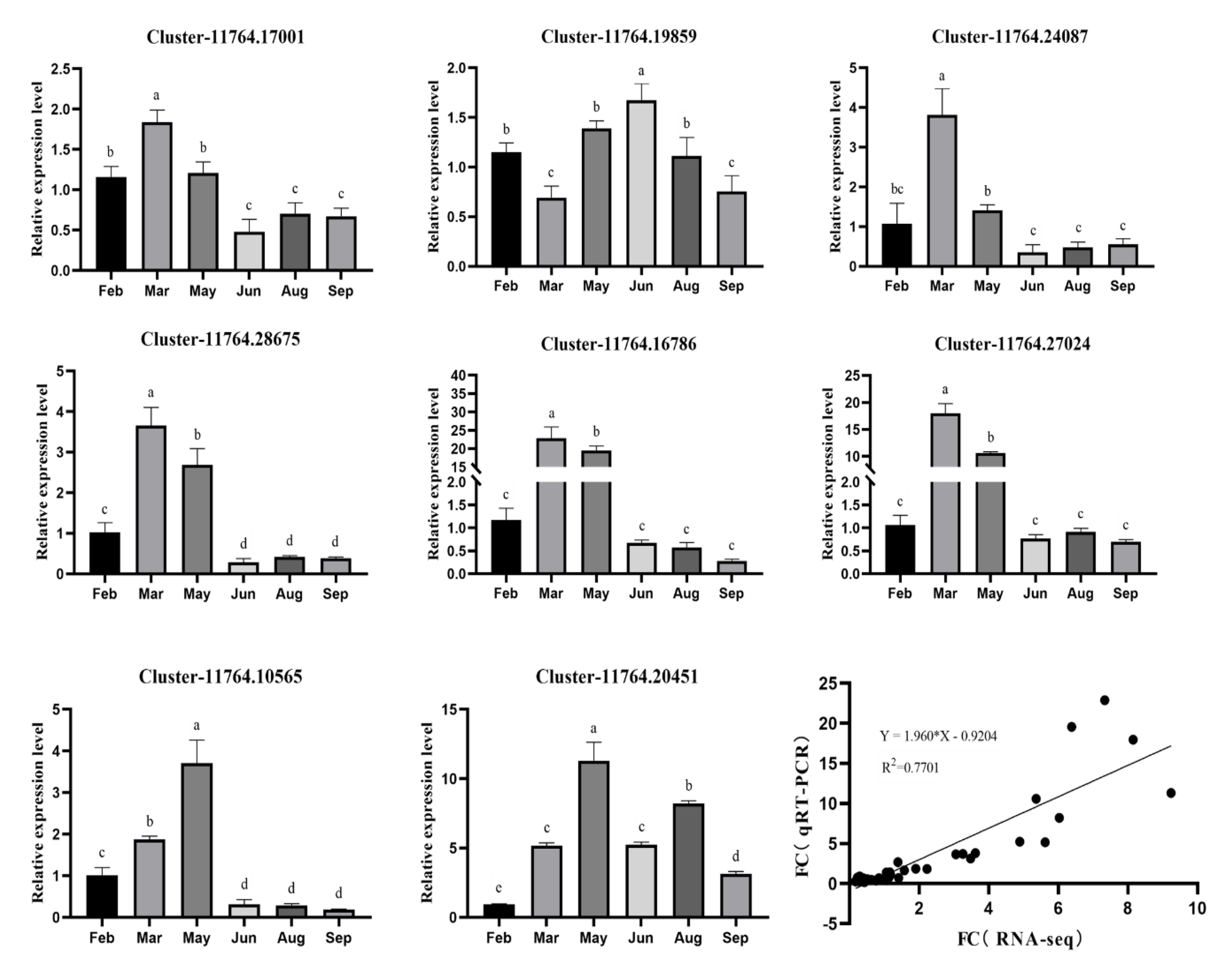

Supplement: Supplementary file 1 [file ijms-24-14976-s001.zip › Supplementary files/Figure S2.jpg]

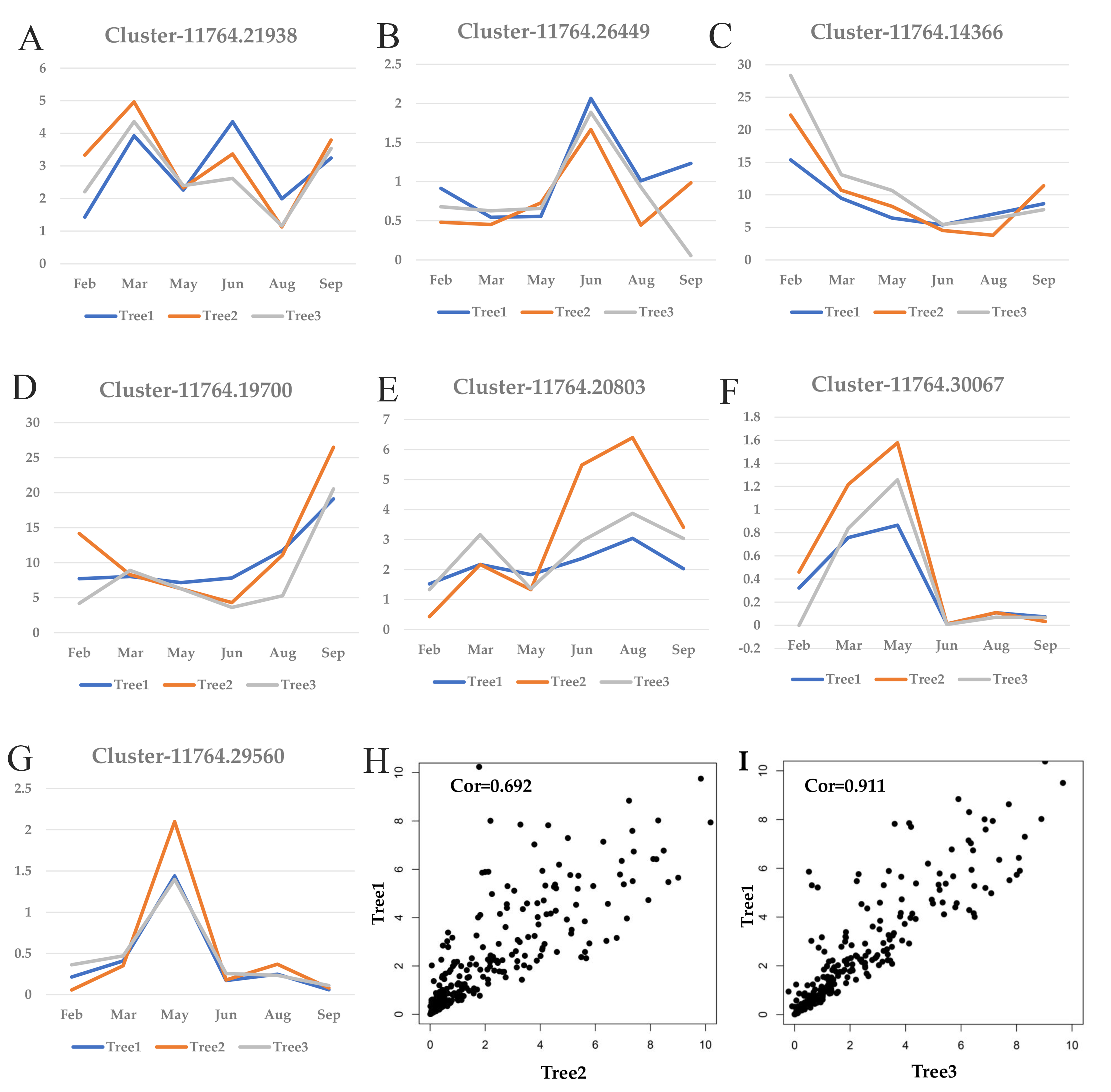

Supplement: Supplementary file 1 [file ijms-24-14976-s001.zip › Supplementary files/Figure S3.jpg]
